# MCI AND CAREGIVING: DYADIC STRESS AND DEPRESSION CONTAGION EFFECTS

**DOI:** 10.1093/geroni/igad104.3464

**Published:** 2023-12-21

**Authors:** Emily Giannotto

**Affiliations:** Emory University, Atlanta, Georgia, United States

## Abstract

Individuals diagnosed with Mild Cognitive Impairment (MCI) remain functionally independent and able to complete instrumental activities of daily living while simultaneously experiencing subjective and objective decline in one or more areas of cognition. Loved ones of individuals with MCI are also impacted and spouses often serve as formal or informal caregivers. Relationships impacted by cognitive impairment experience widespread impacts among cognitive, behavioral, and affective factors within and between individuals. The present study took a novel approach to understanding longitudinal daily parallel patterns of depressive affect, relationship mutuality, sleep, stress, and behavioral problems associated with cognitive impairment. 27 dyads comprised of one spouse with diagnosed Mild Cognitive Impairment (M= 74.6 years, SD= 8.26, 10 female), and the other as a cognitively healthy caregiving spouse (M= 71.4 years, SD= 8.35, 18 female) completed individual nightly reports for two consecutive weeks. Exploratory multilevel modeling revealed significant and complex interactions among spouses’ depressive affect, stress, relationship mutuality, and behavioral problems. Results indicate co-occurring daily effects for depressive affect and stress as contagion effects, with mutuality serving as an important moderator. The impact of mutuality was also significant over and above its role in the interactions. Mutuality seems to act as a buffer for negative affect and stress contagion from one partner to the other across days and from one day to the next. These findings highlight the importance of healthy daily relationship dynamics in mitigating stress and depressive affect contagion among older adults impacted by MCI.

